# Home‐site fidelity and homing behavior of the big‐headed turtle *Platysternon megacephalum*

**DOI:** 10.1002/ece3.7475

**Published:** 2021-05-02

**Authors:** Fanrong Xiao, Rongping Bu, Liu Lin, Jichao Wang, Haitao Shi

**Affiliations:** ^1^ Ministry of Education Key Laboratory for Ecology of Tropical Islands Key Laboratory of Tropical Animal and Plant Ecology of Hainan Province College of Life Sciences Hainan Normal University Haikou People’s Republic of China

**Keywords:** Chelonia, mark–recapture, site fidelity, spatial memory

## Abstract

Site fidelity refers to the restriction of dispersal distance of an animal and its tendency to return to a stationary site. To our knowledge, the homing ability of freshwater turtles and their fidelity is reportedly very low in Asia. We examined mark–recapture data spanning a 4‐year period in Diaoluoshan National Nature Reserve, Hainan Province, China, to investigate the site fidelity and homing behavior of big‐headed turtles *Platysternon megacephalum*. A total of 11 big‐headed turtles were captured, and all individuals were used in this mark–recapture study. The site fidelity results showed that the adult big‐headed turtles (*n* = 4) had a 71.43% recapture rate in the original site after their release at the same site, whereas the juveniles (*n* = 1) showed lower recapture rates (0%). Moreover, the homing behavior results showed that the adults (*n* = 5) had an 83.33% homing rate after displacement. Adult big‐headed turtles were able to return to their initial capture sites (home) from 150 to 2,400 m away and precisely to their home sites from either upstream or downstream of their capture sites or even from other streams. However, none of the juveniles (*n* = 4) returned home, despite only being displaced 25–150 m away. These results indicated that the adult big‐headed turtles showed high fidelity to their home site and strong homing ability. In contrast, the juvenile turtles may show an opposite trend but further research is needed.

## Graphical abstract

We examined mark–recapture data spanning a 4‐year period in Diaoluoshan National Nature Reserve, Hainan Province, China, to investigate the site fidelity and homing behavior of big‐headed turtles (*Platysternon megacephalum*). Our results indicated that the adult big‐headed turtles, but not juveniles, showed a high fidelity to their home site and strong homing ability.

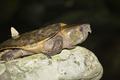

## 1 INTRODUCTION

**FIGURE 1:**
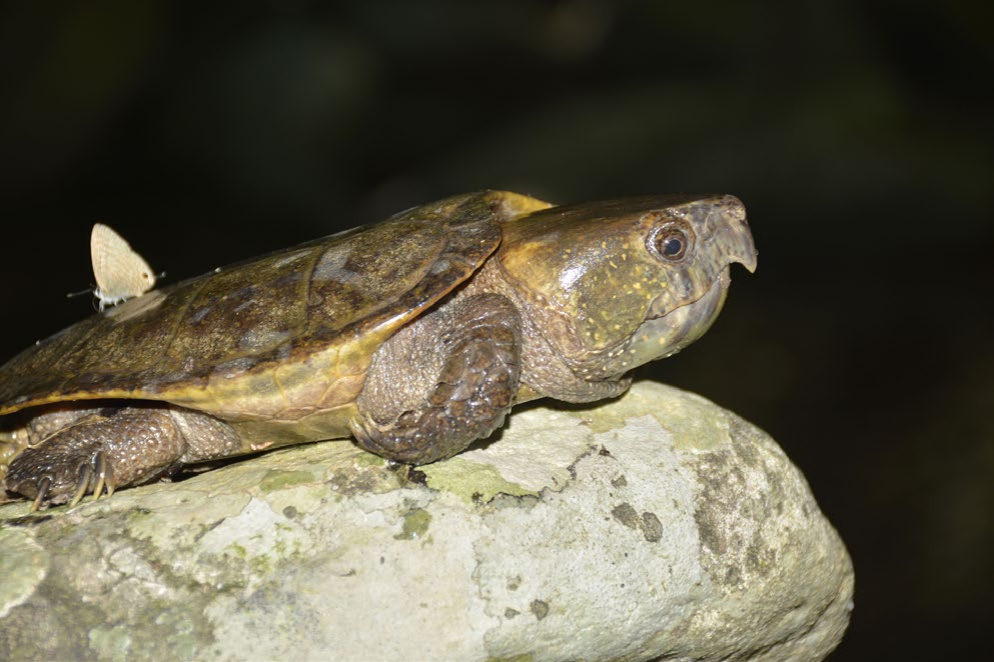
The big‐headed turtle *Platysternon megacephalum* from wild of Hainan Island. Photograph was taken by Fanrong Xiao

Site fidelity is the dispersal distance restriction of an animal and its tendency to return to a stationary site (Switzer, 1993). The most well‐known and well‐documented example of site fidelity is the nest‐site fidelity of egg‐laying animals, for example, turtles, including both sea (Tucker, 2010) and freshwater turtles (Bona et al., 2012). However, to our knowledge, home‐site fidelity, that is, the ability of an animal to remain at its home site without being displaced (Andres & Chambers, 2006), is only exhibited by very few freshwater turtles, and their fidelity is reportedly very low. For example, common musk turtles *Sternotherus odoratus* exhibit only 15% fidelity (recaptured individuals in original site/total individuals) to their home site (Andres & Chambers, 2006). Homing behavior, that is, the ability of a displaced animal (moved from a familiar site to an unfamiliar one) to return to its home site, is another aspect of home‐site fidelity. A few freshwater turtle species, such as the common musk turtle, painted turtle *Chrysemys picta*, spotted turtle *Clemmys guttata*, and red‐eared slider *Trachemys scripta elegans,* have been reported to show homing behavior (Ernst, 1968; Andres & Chambers, 2006). Most freshwater turtle species belong to the order Chelonia, but despite Asia having one of the highest diversity of chelonians worldwide (Mittermeier et al., 2015), there are no reports on the home‐site fidelity and homing behavior of Asian turtle species. Interestingly, we once found that an adult big‐headed turtle *Platysternon megacephalum* (Figure 1), which previously translocated to a different stream in Diaoluoshan National Nature Reserve in April 2015, was recaptured at the original site, that is, the initial capture stream, in May 2016. Considering this, we hypothesized that big‐headed turtles exhibit homing behavior and have high fidelity to their home site. To test this hypothesis, we examined mark–recapture data spanning a 4‐year period in Diaoluoshan National Nature Reserve, Hainan Province, China, to determine whether turtles caught at specific locations were recaptured at their initial capture sites during the same year or subsequent years to investigate their site fidelity. In addition, recapture data for turtles released at their capture sites and those released at sites 25–2400 m away were analyzed to determine whether the turtles exhibited homing behavior.

## 2 MATERIALS AND METHODS

**FIGURE 2:**
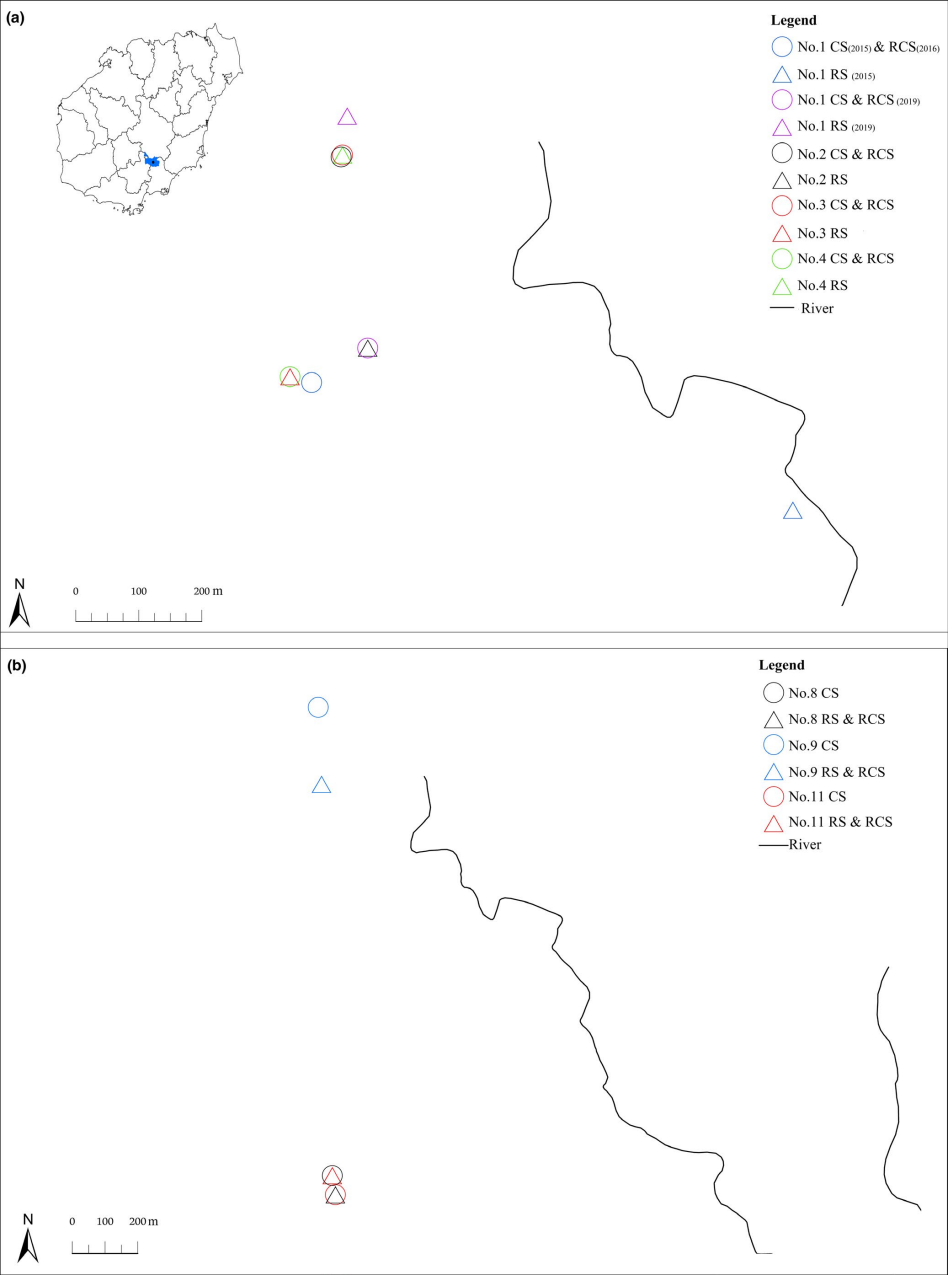
The capture sites (CS), release sites (RS), and recapture sites (RCS) of the adult (A) and the juvenile (B) big‐headed turtles used in the homing behavior experiment. The image in the top left of (A) shows the location of the study site in Diaoluoshan National Nature Reserve (depicted in blue) on Hainan Island, China. The home site of turtle No. 1 differed between the two recaptures because of the destruction of its microhabitat (under a bridge) by human interference in 2018. These sites are in the streams, but these relatively small streams cannot be shown without ready‐made layers

Field studies were conducted in the Diaoluoshan National Nature Reserve, Hainan Province, China, within an altitude range of 500–1050 m a. s. l. from 2015 to 2019 (except 2017). Population density of the big‐headed turtle was 1.65 individuals per km in our study area, and they have been less affected by hunting in recent years under the strict management of the nature reserve (Xiao et al., 2021). Long cylindrical nylon cages (length: 60 cm, diameter: 33 cm; baited with dried fish) were arranged along the stream at an interval of ~30 m. The traps were set for a total of 3 days and checked every morning. After capture, the carapace length of each individual was recorded, and their sex and age‐group were determined. Females and males with mean carapace lengths of more than 100 and 130 mm, respectively, were regarded as adults (Sung et al., 2015a). For identification, captured turtles were uniquely marked on their marginal scutes, with care to avoid injury. Marked individuals were released at their capture sites (original or home sites) or a translocation site for the site fidelity experiment and then recaptured at their original site or release site throughout the year, as well as during subsequent years. Translocation sites were not randomly chosen; they were, instead, the original sites of another turtle (Figure 2) under two presumptions (a) that different individuals of the same species would prefer similar habitats and (b) that the habitats of the translocation sites would be similar in habitat types and characteristics of the original sites. We calculated the recapture rate as the ratio of the times the turtles were recaptured in the original sites and the total time of recapture. This recapture rate was used as an indicator of site fidelity, where a high recapture rate in the original site would indicate a high site fidelity. In the homing behavior experiment, the recapture rate in the original site was used as the homing rate. In addition, the geographic coordinates of the original and translocation sites were determined using a hand‐held global positioning system unit and the trajectory distance along the streams to represent the distance between the original and translocation sites. For adults, the translocation sites were 150–2400 m away (except 35 m for one individual), which is beyond an adult home range distance (97 m; Sung et al., 2015b), whereas juveniles were released 25–150 m away. At the end of the experiment, all cages were removed to avoid harming the local wildlife, and all recaptured individuals were released at their original sites.

## 3 RESULTS

From 2015 to 2019 (except for 2017), a total of 11 big‐headed turtles were captured (*n* = 3 females, *n* = 3 males, and *n* = 5 juveniles). These individuals were used in this mark–recapture study. The site fidelity experiment between 2016 and 2019 revealed that adult big‐headed turtles (*n* = 1 female and *n* = 3 males) showed a high recapture rate in the original site (71.43%, 5 recaptures in the original site/7 total recaptures) after their release at the original site, whereas the juveniles showed lower recapture rates (0%, there were two subsequent trapping efforts after initial trapping for 1 individual but it was never caught again) (Table 1). The homing behavior experiment between 2015 and 2019 revealed that adults (*n* = 3 females and *n* = 2 males) showed a very high homing rate after displacement, that is, as high as 83.33% (5 recaptures in the original site/6 total recaptures; Table 2). Adult big‐headed turtles were able to return to their initial capture sites (home) from 150 to 2,400 m away (Figure 2a) and precisely to their home sites from either upstream or downstream of their capture sites or even from other streams (Table 2). However, none of the juveniles (*n* = 4) returned home, despite only being displaced 25–150 m away (Table 2). In contrast, the release site fidelity (recapture rate in the release site = 3/4 = 75.00%; Figure 2b) was exhibited by juveniles for over 2 months in 2019. These findings indicated that adult big‐headed turtles have a strong homing ability, but juveniles do not.

**TABLE 1:** Home‐site fidelity (recapture rate in the home or original capture site) of big‐headed turtles (*Platysternon megacephalum*)

No.	Age stage	Carapace length (mm)	Sex	Capture date	Capture and release site	Recapture date	Recapture site	Home‐site fidelity
2	Adult	126.95	Female	May 2016	Home	Jul. 2018	Home	71.43% (5/7)
				Jul. 2018	Home	May 2019	Home	
3		167.39	Male	May 2016	Home	Jul. 2018	Home	
				Jul. 2018	Home	May 2019	Home	
4		141.33	Male	May 2016	Home	Jul. 2018	Not found	
						May 2019	Home	
5		131.89	Male	Oct. 2018	Home	May 2019	Not found	
7	Juvenile	85.91	—	May 2016	Home	Jul. 2018	Not found	0% (0/2)
						May 2019	Not found	

Note

“No.” refers to the unique number assigned to each turtle, and “Not found” indicates that the individual was not found in their home site.

**TABLE 2:** Homing rate of big‐headed turtles (*Platysternon megacephalum*)

No.	Age stage	Carapace length (mm)	Sex	Capture date	Capture site	Translocation distance (m)	Release site	Recapture date	Recapture site	Homing rate
1	Adult	149.56	Female	Apr 2015	Home	2,400	Different stream	May 2016	Home	83.33% (5/6)
				May 2019	Home	150	Downstream No. 7 capture site	Jul. 2019	Home	
2		126.95	Female	May 2019	Home	245	Downstream No. 1 capture site	Jul. 2019	Home	
3		167.39	Male	May 2019	Home	438	Different stream No. 4 capture site	Jul. 2019	Home	
4		141.33	Male	May 2019	Home	437	Different stream No. 3 capture site	Jul. 2019	Home	
6		113.07	Female	May 2019	Home	35	Upstream No. 5 capture site	Jul. 2019	Not found	
8	Juvenile	69.40	—	May 2019	Home	25	Upstream No. 11 capture site	Jul. 2019	Release site	0% (0/4);or 75% (3/4) of release site fidelity
9		60.65	—	May 2019	Home	45	Downstream No. 2 capture site	Jul. 2019	Release site	
10		63.42	—	May 2019	Home	150	Downstream No. 1 capture site	Jul. 2019	Not found	
11		63.19	—	May 2019	Home	25	Downstream No. 8 capture site	Jul. 2019	Release site	

Note

“Not found” indicates that the individual was not found in the home site nor the release site. The home sites of turtles with the same numbers here were in the same position as the home sites listed in Table 1. Due to the destruction of the microhabitat of turtle No. 1 in 2018, its home sites in 2015 and 2016 differed from that of 2019.

## 4 DISCUSSION

Our findings provide the first evidence of home‐site fidelity and homing behavior in adult big‐headed turtles. These traits are considered to have fitness advantages in almost all instances because they reinforce the knowledge of the location of food sources, shelters from predators, and the local ecological community (Andres & Chambers, 2006). In the present study, we used the original capture sites as the translocation sites of the other turtles, with the presumptions that (a) the microhabitats of the translocation sites would be similar to those of the home sites of translocated turtles because (b) different individuals of the same species would prefer similar habitats. Therefore, it is assumed that the observed behaviors in the present study were not caused by the unsuitability of the microhabitats of the translocation sites. In contrast, the territoriality of big‐headed turtles (Gong et al., 2019) may explain their high home‐site fidelity and homing rates.

We found that adult big‐headed turtles could find their way back to their home sites after displacement to 150–2400 m away, which is beyond their home range distance (Sung et al., 2015b), even after displacement to different streams. Previous studies have demonstrated that chemical signals (smell), spatial memory (experience), and sun compass navigation play important roles in the navigation of freshwater turtles (Roth & Krochmal, 2015; Yeomans, 1995), whereas sea turtles navigate using a geomagnetic map (Lohmann et al., 2004). The role of chemical signals in the long‐distance orientation of big‐headed turtles can be excluded because they could return to their home sites when displaced to different streams or upstream from their home sites. However, they may use chemical signals to identify their home sites within a range after orientation using other means. Although yearling snapping turtles *Chelydra serpentina* use a geomagnetic compass to orient in a laboratory setting (Landler et al., 2015), there is no strong evidence that freshwater turtles use a geomagnetic map to navigate in natural habitat, and further investigations are needed to determine whether big‐headed turtles have the ability to use geomagnetic navigation. In contrast, some field studies of freshwater turtles in natural habitats had suggested that a sun compass enables freshwater turtles to maintain their orientation when environmental cues were not visible (DeRosa & Taylor, 1978, 1982; Caldwell & Nams, 2006; Krenz et al., 2018; Pappas et al., 2009). Therefore, big‐headed turtles may use sun compasses to find their way home. Moreover, navigation in freshwater turtles has been shown to depend on their spatial memory, with hatchlings having a limited ability that increases with age (Roth & Krochmal, 2015). In the present study, juvenile big‐headed turtles remained in the release sites instead of returning to their previous capture sites, despite the distance between the two sites being very short. Therefore, we also inferred that big‐headed turtles in the tropical forest might rely on spatial memory to navigate back to their home sites. This notion may also explain why only the adult big‐headed turtles, but not the juveniles, exhibited high home‐site fidelity and homing rate.

### 4.1 Conclusion

In conclusion, this study demonstrated high fidelity of adult big‐headed turtles to their home sites and confirmed homing behavior in this species. Further research is needed to clarify the mechanisms underlying these behaviors. However, for juvenile big‐headed turtles, whether they have home‐site fidelity and homing behavior should be judged with caution because of the small sample size and an unclear home range distance, which are limitations of this study. In the juvenile homing behavior experiment, a home range distance within 45 m resulted in the turtles not returning to their original site when translocated no more than 45 m because they were still in their home site range. Thus, the 75% release site fidelity in this study may be indicated instead of their fidelity to the home site.

## CONFLICT OF INTEREST

The authors declare no conflicts of interest.

## AUTHORS’ CONTRIBUTIONS

**Fanrong Xiao:** Conceptualization (Equal); Investigation (Equal); Writing‐original draft (Equal). **Rongping Bu:** Investigation (Equal); Writing‐original draft (Equal). **Liu Lin:** Investigation (Equal). **Jichao Wang:** Writing‐review & editing (Equal); **Haitao Shi:** Conceptualization (Equal); Writing‐review & editing (Equal).

## ETHICS APPROVAL

All fieldwork was conducted in strict accordance with the guidelines of the Animal Research Ethics Committee of Hainan Provincial Education Centre for Ecology and Environment, Hainan Normal University (HNECEE‐2014–002), which conforms to the Law of the People's Republic of China.

## Data Availability

These data are available at https://doi.org/10.5061/dryad.280gb5mpk

## References

[ece37475-bib-0001] Andres, K. M. , & Chambers, R. M. (2006). A test of philopatry by common musk turtles. American Midland Naturalist, 156, 45–51.

[ece37475-bib-0002] Bona, M. , Novotný, M. , Danko, S. , & Burešová, A. (2012). Nest site fidelity in the Slovakian population of the European pond turtle *Emys orbicularis* . Amphib‐reptil, 33, 207–213.

[ece37475-bib-0003] Caldwell, I. R. , & Nams, V. O. (2006). A compass without a map: tortuosity and orientation of eastern painted turtles (*Chrysemys* *picta* *picta*) released in unfamiliar territory. Canadian Journal of Zoology, 84, 1129–1137.

[ece37475-bib-0004] DeRosa, C. T. , & Taylor, D. H. (1978). Sun‐compass orientation in the painted turtle, *Chrysemys picta* (Reptilia, Testudines, Testudinidae). Journal of Herpetology, 12, 25–28.

[ece37475-bib-0005] DeRosa, C. T. , & Taylor, D. H. (1982). A comparison of compass orientation mechanisms in three turtles (*Trionyx spinifer*, *Chrysemys picta*, and *Terrapene carolina*). Copeia, 1982, 394–399.

[ece37475-bib-0006] Ernst, C. H. (1968). Homing ability in the spotted turtle, *Clemmys guttata* (Schneider). Herpetologica, 24, 77–78.

[ece37475-bib-0007] Gong, S. P. , Hua, L. S. , Ge, Y. , & Cao, D. N. (2019). Unique multiple paternity in the endangered big‐headed turtle (*Platysternon megacephalum*) in an ex situ population in South China. Ecology and Evolution, 9, 9869–9877. 31534700 10.1002/ece3.5528 PMC6745651

[ece37475-bib-0008] Krenz, J. D. , Congdon, J. D. , Schlenner, M. A. , Pappas, M. J. , & Brecke, B. J. (2018). Use of sun compass orientation during natal dispersal in Blanding’s turtles: *In* *situ* field experiments with clock‐shifting and disruption of magnetoreception. Behavioral Ecology and Sociobiology, 72, 1–9.

[ece37475-bib-0009] Landler, L. , Painter, M. S. , Youmans, P. W. , Hopkins, W. A. , & Phillips, J. B. (2015). Spontaneous magnetic alignment by yearling snapping turtles: Rapid association of radio frequency dependent pattern of magnetic input with novel surroundings. PLoS One, 10, e0124728. 25978736 10.1371/journal.pone.0124728 PMC4433231

[ece37475-bib-0010] Lohmann, K. J. , Lohmann, C. M. F. , Ehrhart, L. M. , Bagley, D. A. , & Swing, T. (2004). Geomagnetic map used in sea‐turtle navigation. Nature, 428, 909–910. 15118716 10.1038/428909a

[ece37475-bib-0011] Mittermeier, R. A. , Van Dijk, P. P. , Rhodin, A. G. J. , & Nash, S. D. (2015). Turtle hotspots: An analysis of the occurrence of tortoises and freshwater turtles in biodiversity hotspots, high‐biodiversity wilderness areas, and turtle priority areas. Chelonian Conservation and Biology, 14, 2–10.

[ece37475-bib-0012] Pappas, M. J. , Congdon, J. D. , Brecke, B. J. , & Capps, J. D. (2009). Orientation and dispersal of hatchling Blanding’s turtles (*Emydoidea blandingii*) from experimental nests. Canadian Journal of Zoology, 87, 755–766.

[ece37475-bib-0013] Roth, T. C. , & Krochmal, A. R. (2015). The role of age‐specific learning and experience for turtles navigating a changing landscape. Current Biology, 25, 333–337. 25578905 10.1016/j.cub.2014.11.048

[ece37475-bib-0014] Sung, Y. H. , Hau, B. C. H. , & Karraker, N. E. (2015b). Spatial ecology of endangered big‐headed turtles (*Platysternon megacephalum*): Implications of its vulnerability to illegal trapping. Journal of Wildlife Management, 79, 537–543.

[ece37475-bib-0015] Sung, Y. H. , Hau, B. C. H. , Lau, M. W. N. , Crow, P. A. , Kendrick, R. C. , Buhlmann, K. A. , Ades, G. W. J. , & Karraker, N. E. (2015a). Growth rate and an evaluation of age estimation for the endangered big‐headed turtle (*Platysternon megacephalum*) in China. Journal of Herpetology, 49, 99–103.

[ece37475-bib-0016] Switzer, P. V. (1993). Site fidelity in predictable and unpredictable habitats. Evolutionary Ecology, 7, 533–555.

[ece37475-bib-0017] Tucker, A. D. (2010). Nest site fidelity and clutch frequency of loggerhead turtles are better elucidated by satellite telemetry than by nocturnal tagging efforts: Implications for stock estimation. Journal of Experimental Marine Biology and Ecology, 383, 48–55.

[ece37475-bib-0018] Xiao, F. , Bu, R. , Lin, L. , Mueti, J. , Wang, J. , Ye, Z. , & Shi, H. (2021). A survey of freshwater turtles in diaoluoshan nature reserve with conservation implications for the endangered big‐headed turtle. Chelonian Conserv Bi 20(1). (will be published in June, 2021)

[ece37475-bib-0019] Yeomans, S. R. (1995). Water‐finding in adult turtles: Random search or oriented behaviour? Animal Behavior, 49, 977–987.

